# Psychological health of pregnant and postpartum women before and during the COVID-19 Pandemic

**DOI:** 10.1371/journal.pone.0267042

**Published:** 2022-04-14

**Authors:** Yvonne J. Kuipers, Roxanne Bleijenbergh, Laura Van den Branden, Yannic van Gils, Sophie Rimaux, Charlotte Brosens, Astrid Claerbout, Eveline Mestdagh

**Affiliations:** 1 Department of Health and Social Care, School of Midwifery, AP University College, Antwerp, Belgium; 2 School of Health and Social Care, Edinburgh Napier University, Edinburgh, Scotland; 3 Faculty of Medicine and Health Sciences, Antwerp University, Wilrijk, Belgium; VIT University, INDIA

## Abstract

**Background:**

The COVID-19 pandemic is likely to influence psychological health of pregnant and postpartum women.

**Methods:**

We conducted a non-concurrent cross-sectional study among 1145 women living in the Dutch-speaking part of Belgium, 541 pregnant and 604 postpartum women. We measured psychological health with the Whooley questions, Generalized Anxiety Disorder 2-item (GAD-2) and the Edinburgh Postnatal Depression Scale (EPDS) and compared the scores of pregnant and postpartum women before and during the COVID-19 pandemic.

**Results:**

No differences were observed in the Whooley, GAD-2 or EPDS scores among pregnant women. The postpartum total GAD-2 scores before *vs* during the pandemic showed significant differences. Controlling for confounders, we observed a small main positive effect of having an infant during time of COVID-19 (*F*(1.13) = 5.06, *p*.025, *d*.27). The effect was significantly larger for women with (a history of) perinatal psychological problems (*F*(1.12) = 51.44, *p* < .001, *d*.82). Emotional support was significantly related to GAD-2 scores of postpartum women during the pandemic (*F*(1.90) = 35.54, *p* < .001). Postpartum women reported significant higher effects of the pandemic on their behavior compared to pregnant women (*p*.034).

**Conclusion:**

The COVID-19 pandemic seems to have a positive effect on postpartum women during the first year postpartum, in particular for women with (a history of) perinatal psychological problems and for those women who experienced emotional support. The findings suggest that less external stimuli caused by lockdown restrictions might have a positive effect on postpartum women’s emotional wellbeing. The sample consisted of white, educated women in a relationship and information regarding the extent of exposure to adverse COVID-19 consequences was lacking. We relied on self-selection and self-report. The postpartum pandemic sample was small.

## Introduction

The 2019 novel corona SARS-nCoV-2-virus, better known as the Corona VIrus Disease (COVID-19), has become a threat to global health. The onset of COVID-19 was detected in Wuhan City, China in November 2019 and subsequently spread worldwide. On March 11, 2020, the World Health Organization (WHO) declared the COVID-19 disease as a global pandemic [1]. In an effort to control the spread of COVID-19, almost all countries implemented a range of public health and social restrictive measures [2].

### COVID-19 pandemic in Belgium

Belgium introduced the lockdown period on March 13, 2020. The dominant message was to stay at home. Physical activities such as walking, or cycling were allowed and working from home was advised. Social distancing rules had to be respected, otherwise fines would follow. All catering establishments and non-essential shops were closed. Drones spreading announcements were used to remind people to stay indoors and warned people to adhere to the COVID-19 rules and to quarantine measures. People showed extreme hoarding behavior. Travelling abroad was prohibited and the borders with neighboring countries were closed. In May 2020 the lockdown eased; shops, bars and restaurants reopened, and people were allowed to meet up with more people. The first wave ended at the start of the summer holidays and swimming pools, gyms, cinemas, etc., were reopened and travelling within Europe was permitted. At the end of the summer 2020, the second COVID-19 wave hit Belgium, followed by a second lockdown on October 19, 2020. Working from home became mandatory. In December 2020 the regulations eased again and at the end of December 2020 the vaccination campaign kicked off, marking the end of Belgium’s second wave. In March 2021, the third wave hit Belgium although it showed a lower epidemic peak compared to the first two waves. During the pandemic waves, social distancing rules were synchronous with the rising and flattening curves of the waves, allowing meeting less or more people, depending on the number of positive tests and ICU admissions. Between March 2020 and April 2021 (due to workload in the hospitals, the registration was discontinued), 1116 pregnant Flemish mothers had a positive COVID-19 test of which 31 women were admitted on the Intensive Care Unit (ICU). Ten neonates of mothers with COVID-19 were tested positive. There were no maternal deaths as a result from COVID-19. [3]. Pregnant and breastfeeding mothers were added as a priority group to the vaccination campaign [4].

Domestic lockdown and social isolation have proven to be reasonably effective in terms of infection. However, on a psychosocial level isolation carries the risk for the onset of feelings such as uncertainty, fear, and despair as a result of isolation and of economical and job losses [2, 5]. Working from home in combination with (home schooling) children showed to be quite challenging during lockdown. Since the outbreak of COVID-19, stay-at-home orders have caused millions of children to remain out of school or childcare, and thus support from outside the family unit has abruptly reduced. Grandparents were advised to keep distance and, as a result, parents suddenly had solely each other to rely on [2]. In addition, the levels of reported domestic and family violence have increased during the COVID-19 pandemic [6]. Personal emotional wellbeing among the Flemish population wellbeing slightly reduced during the first COVID-19 lockdown period. However, it improved after easing the restrictions, suggesting an association between psychological health and levels of restrictions during the pandemic [7].

### Childbirth during COVID-19

Women during the third trimester or women with underlying conditions might be at risk for critical respiratory illness and admission [8]. Reports of premature birth, premature rupture of membranes and risk of miscarriage associated with COVID-19 remains unclear as information is inconsistent [8–12]. Women reported changes in antenatal care due to the pandemic, including online appointments, cancelled appointments, or not being allowed to bring another person [13–17]. Within the maternity units, strict measures applied to visitors, including own children, but rules regarding the visits of the partner varied per maternity unit [18]. Perinatal women had to adapt rapidly to changing and uncertain circumstances, with scarce information or inconsistent messages and advice from public health bodies concerning antenatal care and support [19]. An emerging trend has been observed towards an increasing number of home births, a shorter stay in the maternity units and an increase of breastfeeding [20, 21]. Some pregnant women during the pandemic changed their mind about place of birth and were more inclined to have an out-of-hospital birth [15, 20]. Despite the great uncertainties brought on by the pandemic, positive impact of the lockdown has also been reported. Postpartum women voiced to enjoy having more privacy and feeling more relaxed because of fewer visitors, experiencing greater partner support and successful breastfeeding [22].

### Psychological aspects of COVID-19 and childbirth

Five main causes of psychological distress during lockdown have been identified: the duration of lockdown, fear of infection, feelings of frustration and boredom, inadequate supplies, and inadequate information [23], leading to repetitive worrying, stress, depression and/or obsessive thoughts [24]. The COVID-19 pandemic added stress to women in the perinatal period and symptoms of anxiety and depression were heightened by social distancing and fear of the virus and the uncertainties associated with COVID-19 [22, 25, 26]. Elevated levels of depression and anxiety have been reported among pregnant women during the COVID-19 period [13, 27–29] and among postpartum women [30, 31], although some studies have reported a decrease of depression among pregnant and postpartum women [32–34]. Psychological health is negatively affected by lockdown restrictions and measures during the COVID-19 epidemic, when compared to non-pandemic figures of perinatal depression and anxiety [17, 35, 36]. Some studies have used retrospective self-reports of women regarding pre-pandemic and pre-lockdown psychological health [31, 36], introducing reporting bias.

Being able to follow individuals based on common features such as region or country, background characteristics or socio-economic status and being exposed to the same pandemic regulations, classified according to exposure status (i.e., exposed, or unexposed to the COVID-19 pandemic and the related lockdown and regulations) is of merit to make a statement whether emotional wellbeing of perinatal women changes during the pandemic [17, 32–34]. This allows to determine if the development of psychological health is different in the exposed or unexposed groups. We aimed to establish and compare women’s antenatal and postpartum psychological health before and during the COVID-19 pandemic, adding to existing evidence.

## Methods

### Design

We conducted a non-concurrent cross-sectional study among women in Flanders (the Dutch-speaking part of Belgium) during any trimester of pregnancy and up to one-year postpartum. Eligible women were 18 years of age or older, with a good comprehension of the Dutch language. We excluded women with illnesses or life-threatening conditions requiring intensive medical support and mothers with children with congenital anomalies, severe pathology, or life-threatening diseases. The data were collected between August 8, 2019, and February 17, 2021, using online self-completed questionnaires (Limesurvey^©^ software).

### Sampling

Before the COVID-19 period and during the pandemic, we recruited participants in a similar way by combining convenience and purposive sampling. Healthcare professionals (midwives, obstetricians, doula’s, antenatal educators, psychologists, health visitors) were informed about the study and were asked to spread flyers and posters announcing the study and inviting potential participants. The posters and flyers included a Uniform Resource Locator (URL) and Quick Response (QR)-code, anonymously redirecting to the questionnaire. Participants were also recruited via social media platforms, allowing snowballing.

### Measures

Three self-report mental health measures were completed alongside socio-demographic information and personal details. Emotional support and practical support of others were measured with one item with a 10-point scale (*1 ‘no support at all’– 10 ‘a lot of support’*). We added four COVID-19 related items enquiring about the effect of the pandemic on thoughts, emotional wellbeing, behavior, and physical health (only completed by participants who completed the questionnaire after March 13, 2020). The four COVID-19 items were each measured with one item using a 10-point scale (*1 ‘not at all’– 10 ‘very much’*). Mental health was self-reported with the Dutch versions of the Whooley questions [37], Generalized Anxiety Disorder 2 item (GAD-2) [38], and the Edinburgh Postnatal Depression Scale (EPDS) [39]. A history of perinatal mental health problems referred to problems during a previous or current pregnancy and/or the postpartum period.

#### Whooley questions

The Whooley items are case-finding questions, identifying potential low mood and loss of interest or pleasure during the past month, are answered either positively (yes) or negatively (no) [40]. A positive response to one or both Whooley items is considered as a positive test [41]. The Dutch version of the Whooley items has been validated among a pregnant population, during first and third trimester of pregnancy. The two items showed good screening utility throughout the course of pregnancy for depression (sensitivity 69–74%, specificity 85–88%, accuracy .80-.85) and for anxiety (sensitivity 58–80%, specificity 85–93%, diagnostic accuracy .83-.86) [42].

#### General Anxiety Disorder 2 items (GAD-2)

The participants who responded positively to the Whooley questions, completed the GAD-2. The GAD-2 is a short version of the seven-item scale, detecting the core symptoms of generalized anxiety in primary care settings. The GAD-2 questions refer to feelings of anxiety over the last two weeks [41]. Responses are scored from 0–3, in seriousness of symptoms. The total score ranges from 0–6. We used a score ≥3 as cut off value for anxiety [43]. Weighted sensitivity of the GAD-2 showed 69% and specificity 91% during early pregnancy [44].

#### Edinburgh Postnatal Depression Scale (EPDS)

The EPDS is a ten-item questionnaire to screen for the likelihood of antenatal and postnatal depression, referring to feelings and thoughts during the past seven days. Responses are scored from 0–3 with a total score range from 0–30 [45]. In this study we used ≥11 as a cut off value for women in the first trimester of pregnancy, ≥10 in second and third trimester of pregnancy and ≥13 for the postpartum period [46, 47]. Per trimester, these cut off scores yield sensitivity (70–79%), specificity (94–97%) in a Dutch-speaking population [48] as well as at various postpartum timepoints six weeks to one year postpartum (sensitivity 66%, specificity 95%) [49].

### Data analysis

First, the cases in the period 3 February–13 March 2020 were deleted. This period was regarded as a transition period between the officially announced start of the pandemic and the first lockdown in Belgium, likely to confound the true effect of the COVID-19 regulations. We split the cases in pre-COVID-19 (before February 3, 2020) and during COVID-19 (after March 13, 2020) groups. Normality of distribution was checked with the Shapiro-Wilk test. When fewer than 10% of the value for a case or for an item were missing, values were imputed with sample means. We calculated descriptive statistics for the sociodemographic and personal characteristics. We summed the scores of the GAD-2 and EPDS and established the cases with scores above cut off values. The strategy for model building was as follows: Mann-Whitney U test and chi-square tests were used to calculate differences between background characteristics and Whooley, GAD-2 and EPDS scores between the study samples (exposed *vs* unexposed women). Significant differences in GAD-2 and/or EPDS scores were followed by a univariate analysis of covariance, to determine whether being pregnant or having a child younger than one year old during a time of COVID-19 regulation produces a significant difference against antenatal and postpartum women before the pandemic started, controlling for differences in sample characteristics. The Kruskal Wallis test was used to examine the differences of the COVID-19 related items’ scores between pandemic pregnant and postpartum women. The *p*-value was set at < .05. An a priori sample size calculation showed that we needed a minimum of 382 participants for both pregnant and postpartum samples (*p* < .05, CI 95%) to make inferences from the sample. The analyses were performed using the Statistical Package for the Social Sciences^©^ (SPSS) version 27.

### Ethical approval and informed consent

The study protocol was reviewed and approved by the Ethics Committee Social and Human Sciences Antwerp (Reference nr. EA_SHW_19_34, 8 August 2019). The questionnaire included a privacy note explaining confidentiality, anonymity, data handling and dissemination of the results. Participants were informed about the study and when they agreed to participate, they provided a written consent. All methods were performed in accordance with the relevant guidelines and regulations (Declaration of Helsinki).

## Results

**Fig 1:**
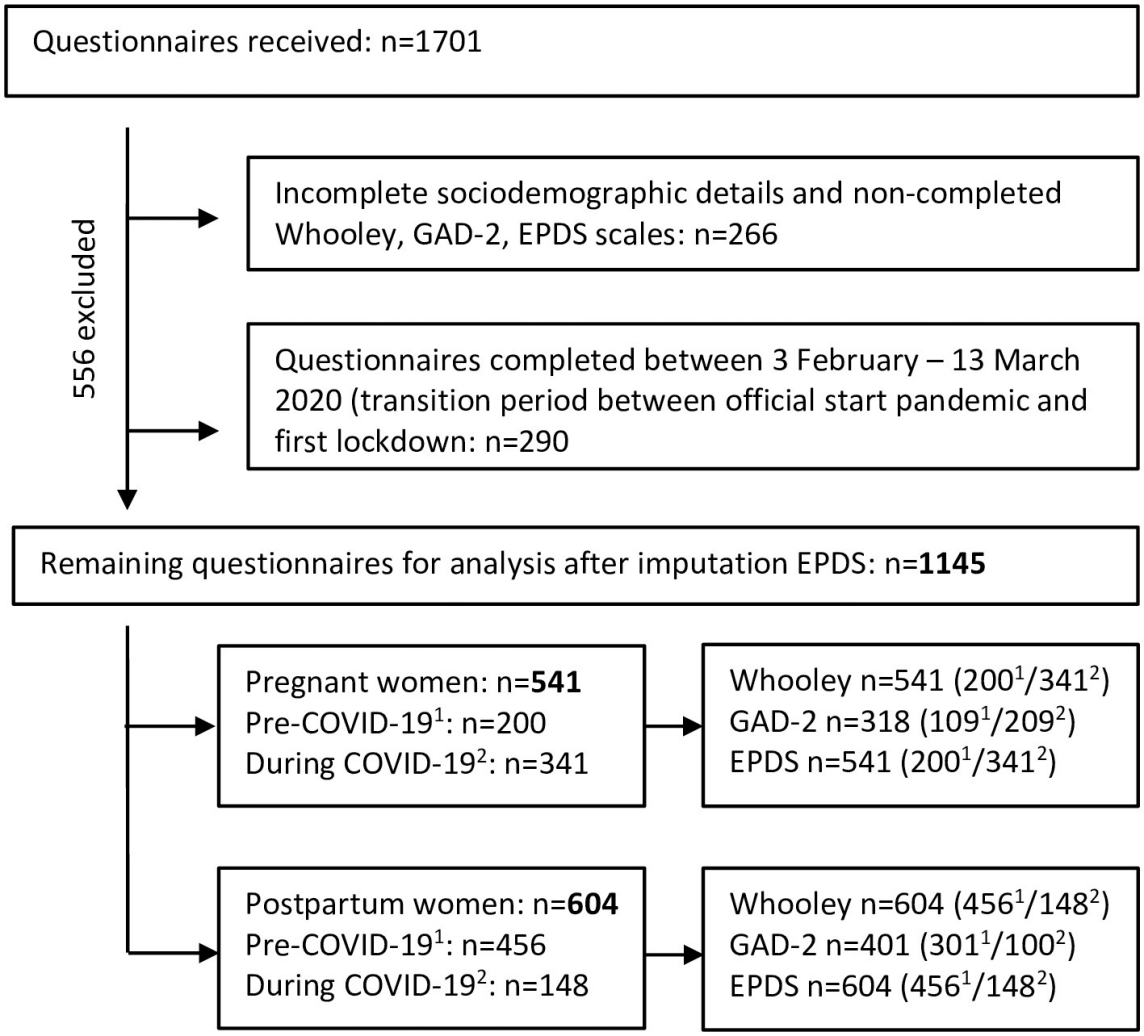
Flow chart. ^1^Data collection period before COVID-19/before 3 February 2020 ^2^Data collection period during COVID-19/after 13 March 2020.

We received 1701 questionnaires of which 266 were removed due to incomplete socio-demographic details and missing values on all the three psychometric scales. Of the remaining 1435, we deleted 290 cases that had completed the questionnaire between February 3 and March 13, 2020 (see Fig 1). The remaining 1145 showed 9.6% (<10%) missing EPDS values which were imputed by the mean EPDS scores. There were no missing values for the Whooley-items, or the GAD-2 scores completed by the participants with positive Whooley answers. The Shapiro-Wilk test showed that the numeracy scores for the following characteristics for the pregnant and the postpartum sample: age, gestational age, length postpartum period, gravidity, parity, emotional and practical support, and the GAD-2 and EPDS scores were significantly non-normal (*p* .006; *p* < .001). Our analysis of maternal perinatal psychological health included 1145 EPDS scores, 1145 Whooley-items and 719 GAD-2 scores.

### Participants

Of the 1145 cases, 541 women were pregnant and 604 were postpartum women. The pregnant sample consisted of 200 pre-COVID-19 participants and 341 women being pregnant during the pandemic. Mann Whitney U and chi-square tests showed no significant differences between the groups (see Table 1). The postpartum sample consisted of 456 pre-COVID-19 participants and 148 postpartum women during the pandemic. We observed that at point of self-report, the pre-COVID-19 participants had given birth significantly longer ago compared to the postpartum participants during the pandemic (*p* < .001). The unexposed postpartum group had significantly more often a history of psychological problems or (a history of) perinatal psychological problems then the exposed group (*p* .029, *p* .01). The exposed postpartum women reported significantly higher levels of emotional support, compared to the pre-COVID-19 unexposed participants (*p* .007) (see Table 2).

**Table 1:** Socio-demographic and personal details pregnant women.

		*Total (n = 541)*	Pre-COVID-19 (n = 200)	During COVID-19 (n = 341)	*p*-value
Age in years	*mean (SD; range)*	*29*.*65 (3*.*88; 18–48)*	29.67 (4.04; 18–48)	29.65 (3.79; 20–44)	0.837^4^
Gestation in weeks	*mean (SD; range)*	*23*.*86 (9*.*93; 1–41)*	24.43 (10.19; 1–40)	23.52 (9.8; 2–41)	0.257^4^
Trimester of pregnancy	*% (n)*				0.830^5^
• First trimester (0–13 weeks)		*19*.*6 (106)*	18.5 (37)	20.2 (69)	
• Second trimester (14–26 weeks)		*36*.*4 (197)*	36 (72)	36.7 (125)	
• Third trimester (27–41+ weeks)		*44 (238)*	45.5 (91)	43.1 (147)	
Gravidity	*mean (SD)*	*1*.*93 (2*.*35)*	2.17 (3.51)	1.79 (1.23)	0.655^4^
Parity	*mean (SD)*	*0*.*59 (0*.*74)*	0.57 (0.69)	0.60 (0.76)	0.992^4^
Job	*% (n)*				
• No paid job		*4*.*3 (23)*	4.5 (9)	4.1 (14)	0.883^5^
• Paid job (1–40 hours/week)		*95*.*7 (518)*	95.5 (191)	95.9 (327)	
Highest level of education^1^	*% (n)*				0.084^5^
• Low level		*0*.*7 (4)*	1.5 (3)	0.3 (1)	
• Medium level		*19*.*5 (105)*	19.5 (39)	19.4 (66)	
• High level		*79*.*8 (432)*	79 (158)	80.3 (274)	
Ethnicity	*% (n)*				0.563^5^
• Belgium		*95*.*2 (515)*	94.5 (189)	95.6 (326)	
• Other		*4*.*8 (26)*	5.5 (11)	4.4 (15)	
Relational status	*% (n)*				0.561^5^
• Married/co-habiting		*95*.*6 (517)*	95 (190)	95.9 (327)	
• Living Apart Together		*0*.*6 (3)*	1 (2)	0.3 (1)	
• Single		*3*.*9 (21)*	4 (8)	3.8 (13)	
Emotional support^2^	*mean (SD)*	*7*.*94 (1*.*59)*	8.01 (1.58)	7.90 (1.61)	0.493^4^
Practical support^2^	*mean (SD)*	*7*.*59 (1*.*80)*	7.66 (1.81)	7.55 (1.80)	0.437^4^
Personal history psychological problems	*% (n)*	*33*.*3 (180)*	34 (68)	32.8 (112)	0.783^5^
(History) perinatal psychological problems	*% (n)*	*15*.*7 (85)*	18 (36)	14.4 (49)	0.263^5^
Currently receiving professional help	*% (n)*	*26*.*2 (142)*	29 (58)	24.6 (84)	0.101^5^
The Covid-19 pandemic influences my^3^					
• Daily thoughts	*mean (SD; range)*			6.57 (1.82; 1–10)	
• Daily emotions	*mean (SD; range)*			5.83 (2.05; 1–10)	
• Daily behaviour	*mean (SD; range)*			6.62 (2.12; 1–10)	
• Physical health	*mean (SD; range)*			3.85 (2.05; 1–10)	

^*1*^*Low level*: elementary, pre-vocational secondary education; *Medium level*: vocational secondary education (preparing for higher education); *High level*: secondary education preparing for Bachelor(-equivalent), Master(-equivalent), university.

^*2*^ *Score*: *1* (no support at all)– 10 (a lot of support).

^*3*^ *Score*: 1 (not at all)– 10 (very much).

^4^ Mann-Whitney U test.

^5^ Chi-Square test.

**Table 2:** Socio-demographic and personal details postpartum women.

		*Total (n = 604)*	Pre-COVID-19 (n = 456)	During COVID-19 (n = 148)	*p*-value
Age in years	*mean (SD; range)*	*30*.*51 (3*.*97; 19–47)*	30.53 (4.06; 19–47)	30.44 (3.7; 20–41)	0.624^5^
Weeks postpartum	*mean (SD; range)*	*22*.*2 (15*.*14; 0–52)*	23.76 (14.91; 0–52)	16.66 (14.64; 0–52)	**<0.001** ^5^
Gravidity	*mean (SD; range)*	*1*.*91 (1*.*96)*	1.95 (2.17)	1.80 (1,03)	0.928^5^
Parity	*mean (SD; range)*	*1*.*49 (0*.*72)*	1.46 (0.70)	1.55 (0,76)	0.240^5^
Method of birth	*% (n)*				0.151^6^
• Spontaneous vaginal birth		*65*.*2 (394)*	62.7 (286)		
• Instrumental birth^1^		*12*.*6 (76)*	13.4 (61)		
• Operative birth^1^		*22*.*2 (134)*	23.9 (109)		
Job	*% (n)*				0.526^6^
• No paid job		*4*.*5 (27)*	4.2 (19)	5.4 (14)	
• Paid job (1–40 hours/week)		*95*.*5 (577)*	95.8 (437)	94.6 (140)	
Highest level of education ^2^					0.715^6^
• Low level		*1 (6)*	1.1 (5)	0.7 (1)	
• Medium level		*19*.*4 (117)*	19.7 (90)	18.2 (27)	
• High level		*79*.*6 (481)*	79.2 (361)	81.1 (120)	
Ethnicity	*% (n)*				0.743^6^
• Belgium		*94 (568)*	93.9 (428)	94.6 (140)	
• Other		*6 (36)*	6.1 (28)	5.4 (8)	
Relational status	*% (n)*				0.497^6^
• Married/co-habiting		*94*.*9 (573)*	94.5 (431)	95.9 (142)	
• Living Apart Together		*0*.*7 (4)*	0.9 (4)		
• Single		*4*.*4 (27)*	4.6 (21)	4.1 (6)	
Emotional support^3^	*mean (SD)*	*7*.*36 (1*.*71)*	7.24 (1.75)	7.72 (1.53)	**0.007** ^5^
Practical support^3^	*mean (SD)*	*7*.*25 (1*.*97)*	7.25 (1.96)	7.24 (2)	0.918^5^
Personal history psychological problems	*% (n)*	*32*.*3 (195)*	34.6 (158)	25 (37)	**0.029** ^6^
(History) perinatal psychological problems	*% (n)*	*26*.*3 (159)*	28.9 (132)	18.2 (27)	**0.01** ^6^
Currently receiving professional help	*% (n)*	*23*.*5 (142)*	25.9 (118)	16.2 (24)	0.227^6^
The Covid-19 pandemic influences my^4^					
• Daily thoughts	*mean (SD; range)*			6.79 (1.94; 1–10)	
• Daily emotions	*mean (SD; range)*			5.99 (2.12; 1–10)	
• Daily behaviour	*mean (SD; range)*			7.06 (2.1; 1–10)	
• Physical health	*mean (SD; range)*			3.72 (1.87; 1–9)	

^*1*^ *Instrumental birth*: *v*entouse or forceps; *Operative birth*: primary (planned) and secondary (emergency) caesarean section.

^*2*^*Low level*: elementary, pre-vocational secondary education; *Medium level*: vocational secondary education (preparing for higher education); *High level*: secondary education preparing for Bachelor(-equivalent), Master(-equivalent), university.

^*3*^ *Score*: *1* (no support at all)– 10 (a lot of support).

^*4*^ *Score*: 1 (not at all)– 10 (very much).

^5^ Mann-Whitney U test.

^6^ Chi-Square test.

### Maternal perinatal psychological health

#### Psychological health during pregnancy

We analyzed the Whooley-items and the EPDS scores of 541 pregnant women. The participants who responded positively to one or two of the Whooley-items, completed the GAD-2 (n = 318). Table 3 shows no significant differences between the number of positive Whooley scores, the total GAD-2 scores or number of scores above cut-off value of the 109 pre-COVID-19 participants and the 209 women being pregnant during the pandemic. There were no differences observed between the EDS total scores or the number of scores above cut-off value of the 200 pre-COVID-19 participants and the 341 women being pregnant during the pandemic. The GAD-2 showed acceptable internal consistency (α .78) and the EDS showed good internal consistency in the pregnant sample (α .87).

**Table 3:** Psychological health pregnant women.

		*Total (n = 541)*	Pre-COVID-19 (n = 200)	During COVID-19 (n = 341)	*p*-value
Whooley-item 1 and/or 2	*% (n)*	*(58*.*7) 318*	(54.5) 109	(61.3) 209	0.151^2^
GAD-2 total score	*mean (SD; range)*	*2*.*18 (1*.*47; 0–6)*	1.98 (1.33; 0–6)	2.29 (1.53; 0–6)	0.157^1^
GAD-2 above cut-off value	*% (n)*	*24*.*8 (79)*	24.8 (27)	24.9 (52)	0.983^2^
EPDS total score	*mean (SD; range)*	*8*.*01 (4*.*82; 0–27)*	7.76 (4.86; 0–23)	8.16 (4.79; 0–27)	0.221^1^
EPDS above cut-off value	*% (n)*	*33*.*1 (179)*	30 (60)	34.9 (119)	0.243^2^

^1^ Mann-Whitney U test.

^2^ Chi-Square test.

#### Postpartum psychological health

We analyzed the Whooley-items and the EPDS scores of 604 postpartum women. The participants who responded positively to one or two of the Whooley-items, completed the GAD-2 (n = 401). Table 4 shows significant differences between the total GAD-2 and scores of the 109 pre-COVID-19 participants and the 209 postpartum women during the pandemic. There were no differences observed between the number of positive Whooley scores, GAD-2 scores above cut-off value of the 109 pre-COVID-19 participants and the 209 women being pregnant during the pandemic. There were no differences observed between the EPDS total scores or EPDS scores above cut-off value of the 200 pre-COVID-19 participants and the 341 postpartum women during the pandemic. The GAD-2 showed acceptable internal consistency (α .79) and the EPDS showed good internal consistency in the postpartum sample (α .89).

**Table 4:** Psychological health postpartum women.

		*Total (n = 604)*	Pre-COVID-19 (n = 456)	During COVID-19 (n = 148)	*p*-value
Whooley-item 1 and/or 2	*% (n)*	*(66*.*4) 401*	(66) 301	(67.6) 100	0.727^2^
GAD-2 total score	*mean (SD; range)*	*2*.*18 (1*.*47; 0–6)*	2.44 (1.69; 0–6)	2.01 (1.48; 0–6)	**0.045** ^1^
GAD-2 above cut-off value	*% (n)*	*29*.*8 (119)*	32 (96)	23 (52)	0.088^2^
EPDS total score	*mean (SD; range)*	*9*.*37 (5*.*29; 0–28)*	9.53 (5.48; 0–29)	8.87 (4.63; 1–21)	0.368^1^
EPDS above cut-off value	*% (n)*	*22*.*4 (135)*	23.2 (106)	19.6 (29)	0.354^2^

^1^ Mann-Whitney U test.

^2^ Chi-Square test.

As shown in Table 4, significant differences between pre and during COVID-19 GAD-2 scores were only observed in the postpartum sample. Analysis of Variance (ANOVA) showed unequal sizes of pre-COVID-19 postpartum women and women with children up to one year of age during the pandemic (*F*(1.39) = 5.06, *p* .03). The squared deviation of the postpartum GAD-2 mean scores was 0.87. We performed a univariate analysis with the GAD-2 total scores as the dependent variable and time period (i.e., pre or during COVID-19) as our fixed-factor. Levene’s test was not significant (*F*(1.39) = 1.46, *p* .23). We added (a history of) psychological problems in general and perinatal psychological problems as random factors and length of postpartum period and emotional support as covariates. There were no outliers or multicollinearity between factors and covariates. We observed a small main positive effect of having a child during time of COVID-19 (*F*(1.13) = .5.06, *p* .025, *d* .27), indicating that GAD-2 scores among postpartum women were significantly higher before (*M* = 2.44, *SE* = .09) than during the pandemic (*M* = 2.01, *SE* = .16). We observed a large positive effect of the COVID-19 among postpartum women with (a history of) perinatal psychological problems during the pandemic (pre-COVID-19 GAD-2 *M* = 3.19, *SD* 1.79; during COVID-19 *M* = 1.87, *SD* 1.37) (*F*(1.12) = 51.44, *p* < .001, *d* .82). We did not observe a significant effect among women with (a history of) non-perinatal related psychological problems. The covariate emotional support was significantly related to the GAD-2 scores of postpartum women before or during the pandemic (*F*(1.90) = 35.54, *p* < .001). No significant relationship was observed with length of postnatal period, suggesting that GAD-2 scores were not significantly different between women who had recently given birth or a longer time ago during the first year postpartum. Kruskal Wallis showed that the pandemic had a significant higher effect on the behavior of postpartum women when compared to pregnant women (*p* .034) but not on their thoughts (*p* .25), emotions (*p* .45) or on their physical health (*p* .49).

## Discussion

The COVID-19 pandemic has been conceived as a major risk factor for depressive and anxiety symptoms for pregnant and postpartum women [25, 35]. Because antenatal and postnatal mental health should be prioritized in facing current ongoing pandemic, the aim of this study was to examine the impact of COVID-19 on mental health in pregnant and postpartum women compared with pre-COVID-19 women. We observed differences in women’s self-reports of the Whooley items, depression and of anxiety during pregnancy before and during the COVID-19 pandemic—suggesting a decrease of psychological health during the pandemic among pregnant women, although our results did not reach significance. Social isolation is known to affect psychological wellbeing in pregnant women [17, 27], which might explain the change of direction in antenatal psychological health before and during the pandemic. Our findings of postpartum women showed a different direction of change. Before the COVID-19 pandemic, postpartum women experienced higher levels of anxiety compared to postpartum women during the pandemic, in particular women with (a history of) perinatal psychological problems. A similar direction of change has been reported about postpartum depression but not regarding postpartum anxiety [32, 34]. Our results suggest that the lockdown and restrictions due to the pandemic had a positive effect on the emotional wellbeing of postpartum women in contrast to the study of Ollivier and colleagues [17]. In addition, our results showed that emotional support had a mediating effect on psychological symptoms of postpartum women. The postpartum women in our study reported significant behavioral changes during the pandemic when compared to women before COVID-19. This might be explained by higher levels of resilience which are likely to moderate the consequences of a pandemic [50].

There are several possible explanations for the differences in psychological health in postpartum psychological health of postpartum women before and during the pandemic. Firstly, we observed a significant difference in the postpartum period at point of completing the questionnaire between the pre-COVID-19 group and the postpartum women during the pandemic. The women during the pandemic had given birth more recently compared to the pre-pandemic participants, implying that the women that completed the questionnaire during the pandemic had been pregnant during the pandemic. Because of little or inconsistent available information about risks for pregnant women, these postpartum women might have been so relieved that their baby was healthy, positively affecting their levels of anxiety. During the social isolation period of the COVID-19 pandemic, it might be that women received more support from their partner. In our study this applied to emotional support. Due to restrict visiting, social distancing rules and other regulations affecting daily activities, out of the house activities and going out or having visitors, women were less disturbed by external stimuli compared to the pre-COVID-19 women—providing the opportunity having more time to focus on (a life with) a baby, facilitating maternal-child attachment. It is known that a maternal-child attachment buffers postpartum symptom of anxiety pandemic [51]. Our findings therefore suggest that the pandemic positively influences bonding. In a study of the general population, participants reported slightly less fatigue than those pre-COVID-19 [52]. Poor sleep quality is associated with stress and postnatal depression. Reduced out-of-home commitments have given the average person more time to relax, warding off fatigue [53]. This might explain the better mental health of our postpartum population. Fewer social obligations and pressures give new parents peace of mind and time to get used to parenthood. There has been much public and academic discussion of the possible negative effects of lockdown [52]. The majority of studies on maternal mental health and COVID-19 contradict our results, the differences may be partially due the differences of the severity of lockdown measures, varying between countries. Governmental regulations have a protective effect against anxiety, facilitating an opportunity for people to increase social cohesion and connection [52]. Belgium was in lockdown for parts of the pandemic but there was no mandatory quarantine. A lockdown is indirectly associated with mental health through increased perceived social support, while mandatory quarantine is strongly associated with poor mental health status through decreased perceived social support [54]. A study conducted in Ireland reported that pregnant women improved their relationships with their partners by talking more, exercising together, and sharing tasks during lockdown. In addition, many hospitals and midwifery practices launched different online courses to offer emotional support, e.g., online information sessions were organized about childbirth, breastfeeding, maternal mental health issues and Question & Answer-sessions about COVID-19 [14, 16, 55]. These aspects might have contributed to low levels of anxiety among our postpartum pandemic participants but are worth to consider in postpartum care, emphasizing privacy in hospital wards and retreating in the home situation.

Our study shows strengths and limitations. This study adds to the existing reports about the mental health response of pregnant and postpartum women during COVID-19 pandemic and to make a comparison with women during pre-COVID-19. This study provided the opportunity to compare the prevalence of anxiety and depressive symptoms before and after the national declaration of the COVID-19 pandemic, with similarity in restrictions compared to other countries [14, 17–21, 25–27, 32–36]. A strength of the study is the use of standardized mental health instruments and a systematic recruitment of an unselected population and the fact that we conducted the study during the three waves of the pandemic, controlling for the effect of the initial shock, uncertainty, adjusted care management and measures related to the first wave but also for seasonality effects. In our study the pre-COVID sample reported about psychological wellbeing before lockdown, restricted physical and social activity, reducing bias, in contrast to the studies of Davenport [36] and Fallon [31], where women self-reported on their pre-COVID emotional wellbeing status in retrospect. A limitation of our study is that the sample consisted mainly of white, highly educated women in relationships and having jobs with no major medical issues. It might be that our participants were more privileged in their home and living circumstances (e.g., space in the home, access to a garden, little financial worries) as it is known that these aspects positively affect maternal experiences and in turn, psychological health [22]. Our sampling method might have resulted in selection bias, possibly attracting participants more inclined to engage with the study and complete the measures. Selection bias might have contributed to under or overrepresenting maternal psychological health problems during the COVID-19 pandemic, although similar findings that the pandemic improved some women’s postpartum mood have been reported [32–34]. We excluded women with severe medical pathology and mothers with children with life-threatening conditions, which likely reduced the outcome variable [56, 57]. Another limitation of our study is that we had no information regarding the extent of exposure to the adverse consequences of COVID-19 and we had limited information about women’s COVID-19 related stressors, such as, had they been infected, had they or significant others been ill, lost family or friends due to COVID-19, were they vaccinated? It could be that the time of completing the questionnaire influenced women’s answers, i.e., was it during strict lockdown or at a moment when restrictions had eased [7]. All these COVID-19 related issues are known to play a role in emotional responses to disaster such as a pandemic [20, 58]. Although our study’s sample size was sufficient to generalize findings to the general population of Flemish childbearing women, the postpartum pandemic sample was small. Future studies should include larger sample sizes. In our study we relied on a self-report, although the Whooley questions, GAD-2 and EPDS are internationally recommended screening measures and report the likelihood of depression and anxiety [42, 44, 49]. Our findings are therefore an indication of perinatal mental health but do not have a diagnostic value. Additionally, the postpartum sample completed the survey at different time points throughout the first year postpartum. For future research, it would be of interest to differentiate between early postpartum women (i.e., first six to 12 weeks postpartum) and women later in the postpartum, examining whether the pandemic regulations have a different sub-sample effect. Because of the self-selective nature of our study, response bias might have been introduced. The generalizability of our findings is limited to similar populations of childbearing women, although we believe that the findings are of interest to countries with similar COVID-19 lockdown regulations as described for the Belgium population.

## Conclusion

The antenatal and postpartum period are particularly vulnerable times for women. Even for healthy women, a pregnancy can be overwhelming and may lead to new symptoms of anxiety—especially during a pandemic. This study overall reports no significant effect of the COVID-19 pandemic on depression and anxiety of pregnant women when compared to pre-pandemic women, which seems reassuring in terms of overall perinatal mental health during COVID-19. The restrictions associated with the COVID-19 pandemic even seem to have a positive effect on psychological health of postpartum women, on anxiety and even more specifically for women with a history of perinatal psychological problems. Emotional support has a mediating effect on postpartum psychological health. Postpartum women reported a behavioral change during COVID-19, suggesting that lockdown restrictions might have a positive effect on postpartum women’s emotional wellbeing. More studies should be done to shed more light on psychological health of pregnant and postpartum during the COVID-19 pandemic. For future studies it would of merit to incorporate larger perinatal subsamples of exposed and unexposed COVID-19 women, to assess the extent to exposure of adverse COVID-19 consequences and to consider the postpartum time point of measure.

## Abbreviations

ANOVA: Analysis of Variance
CI: Confidence Interval
COVID: Corona VIrus Disease
EPDS: Edinburgh Postnatal Depression Scale
GAD-2: Generalized Anxiety Disorder 2-item
ICU: Intensive Care Unit
M: Mean
NICU: Neonatal Intensive Care Unit
QR: Quick Response
SD: Standard Deviation
SE: Standard Error
SPSS: Statistical Package for the Social Sciences
URL: Uniform Resource Locator

## References

[1] Hessami K, Romanelli C, Chiurazzi M, Cozzolino M. COVID-19 pandemic and maternal mental health: a systematic review and meta-analysis. The Journal of Maternal-Fetal & Neonatal Medicine. 2020:1–8. doi: 10.1080/14767058.2020.1843155 33135523

[2] Guo J, De Carli P, Lodder P, Bakermans-Kranenburg MJ, Riem MME. Maternal mental health during the COVID-19 lockdown in China, Italy, and the Netherlands: a cross-validation study. Psychol Med. 2021:1–11. doi: 10.1017/s0033291720005504 33436133 PMC7844185

[3] B-OSS. 2021. A nationwide obstetric surveillance system to identify and analyze rare disorders/ complications of pregnancy in Belgium. SPE. https://www.b-oss.be/covid-results (assessed 9 February 2022).

[4] Sciensano. 2021. COVID-19. Epistat. Brussels. https://epistat.wiv-isp.be/covid/ (assessed 9 February 2021).

[5] Wang C, Pan R, Wan X, Tan Y, Xu L, Ho CS, Ho RC. Immediate Psychological Responses and Associated Factors during the Initial Stage of the 2019 Coronavirus Disease (COVID-19) Epidemic among the General Population in China. International Journal of Environmental Research and Public Health. 2020; 17(5):1729. https://www.mdpi.com/1660-4601/17/5/1729 doi: 10.3390/ijerph17051729 32155789 PMC7084952

[6] Roesch E, Amin A, Gupta J, García-Moreno C. Violence against women during covid-19 pandemic restrictions. *BMJ*. 2020; 369:m1712. doi: 10.1136/bmj.m1712 32381644 PMC7202944

[7] Beutels P, Hens N, Pepermans K, Van Damme P. De Grote Coronona studie. Universiteit Antwerpen, UHasselt, KU Leuven. 2020. https://corona-studie.shinyapps.io/corona-studie/?full=1 (assessed 13 March 2021).

[8] Boushra MN, Koyfman A, Long B. COVID-19 in pregnancy and the puerperium: A review for emergency physicians. The American Journal of Emergency Medicine. 2021; 40:193–198. doi: 10.1016/j.ajem.2020.10.055 33162266 PMC7605788

[9] Adhikari EH, Moreno W, Zofkie AC, MacDonald L, McIntire D, Collins R. Pregnancy Outcomes Among Women With and Without Severe Acute Respiratory Syndrome Coronavirus 2 Infection. *JAMA Netw Open*. 2020; 3(11):e2029256. doi: 10.1001/jamanetworkopen.2020.29256 33211113 PMC7677755

[10] Akhtar H, Patel C, Abuelgasim E, Harky A. COVID-19 (SARS-CoV-2) Infection in Pregnancy: A Systematic Review. Gynecol Obstet Invest. 2020; 85:295–306. doi: 10.1159/000509290 32728006 PMC7490507

[11] Almeida M, Shrestha A, Stojanac D, Miller LJ. The impact of the COVID-19 pandemic on women’s mental health. Arch Womens Ment Health. 2020; 23:741–748. doi: 10.1007/s00737-020-01092-2 33263142 PMC7707813

[12] Topalidou A, Thomson G, Downe S. Covid-19 and maternal and infant health: Are we getting the balance right? A rapid scoping review. The Practising Midwife. 2020; 23(7). doi: 10.1101/2020.03.30.20047969

[13] Lebel C, MacKinnon A, Bagshawe M, Tomfohr-Madsen L, Giesbrecht G. Elevated depression and anxiety symptoms among pregnant individuals during the COVID-19 pandemic. Journal of Affective Disorders. 2020; 277:5–13. doi: 10.1016/j.jad.2020.07.126 32777604 PMC7395614

[14] Baumann S, Gaucher L, Bourgueil Y, Saint-Lary O, Gautier S, Rousseau A. Adaptation of independent midwives to the COVID-19 pandemic: A national descriptive survey. Midwifery. 2021; 94:102918. doi: 10.1016/j.midw.2020.102918 33418511 PMC7762837

[15] Fan S, Guan J, Cao L, Wang M, Zhao H, Chen L. Psychological effects caused by COVID-19 pandemic on pregnant women: A systematic review with meta-analysis. Asian Journal of Psychiatry. 2021; 56:102533. doi: 10.1016/j.ajp.2020.102533 33418283 PMC7833174

[16] Montagnoli C, Zanconato G, Ruggeri S, Cinelli G, Tozzi AE. Restructuring maternal services during the covid-19 pandemic: Early results of a scoping review for non-infected women. Midwifery. 2021; 94:102916. doi: 10.1016/j.midw.2020.102916 33412360 PMC7832106

[17] Olivier R, Aston M, Price S, Sim M, Benoit B, Joy P. Mental health & parental concerns during COVID-19: The experiences of new mothers amidst social isolation. Midwifery. 2021; 94:102902. doi: 10.1016/j.midw.2020.102902 33421662 PMC9756383

[18] Vasilevski V, Sweet L, Bradfield Z, Wilson AN, Hauck Y, Kuliukas L. Receiving maternity care during the COVID-19 pandemic: Experiences of women’s partners and support persons. Women and Birth. 2021. doi: 10.1016/j.wombi.2021.04.012 33941497 PMC8075817

[19] Davis-Floyd R, Gutshow K, Schwartz DA. Pregnancy, birth and the COVID-19 pandemic in the United States. Medical Anthropology. 2020; 39(5):413–427. doi: 10.1080/01459740.2020.1761804 32406755

[20] Moyer CA, Compton SD, Kaselitz E, Muzik M. Pregnancy-related anxiety during COVID-19: a nationwide survey of 2740 pregnant women. Arch Womens Ment Health. 2020; 23:757–765. doi: 10.1007/s00737-020-01073-5 32989598 PMC7522009

[21] Nelson A, Romanis EC. The medicalisation of childbirth and access to homebirth in the UK: Covid-19 and beyond. Medical Law Review. 2021; (0):1–27. doi: 10.1093/medlaw/fwab040 34668011 PMC8574542

[22] Brown A, Shenker N. Experiences of breastfeeding during COVID-19: Lessons for future practical and emotional support. Matern Child Nutr. 2021;17. doi: 10.1111/mcn.13088 32969184 PMC7537017

[23] Brooks SK, Webster RK, Smith LE, Woodland L, Wessely S, Greenberg N. The psychological impact of quarantine and how to reduce it: rapid review of the evidence. The Lancet. 2020; 395(10227):912–920. doi: 10.1016/S0140-6736(20)30460-8 32112714 PMC7158942

[24] Mucci F, Mucci N, Diolaiuti F. Lockdown and isolation: psychological aspects of COVID-19 pandemic in the general population. Clinical Neuropsychiatry. 2020; 17 (2):63–64. doi: 10.36131/CN20200205 34908969 PMC8629090

[25] Thapa SB, Mainali A, Schwank SE, Acharya G. Maternal mental health in the time of the COVID-19 pandemic. Acta Obstet Gynacol Scan. 2020; 99:817–818. doi: 10.1111/aogs.13894 32374420 PMC7267371

[26] Osborne LM, Kimmel MC, Surkan PJ. The Crisis of Perinatal Mental Health in the Age of Covid-19. Maternal and Child Health Journal. 2021; 25(3):349–352. doi: 10.1007/s10995-020-03114-y 33543374 PMC7861156

[27] Durankuş F, Aksu E. Effects of the COVID-19 pandemic on anxiety and depressive symptoms in pregnant women: a preliminary study. The Journal of Maternal-Fetal & Neonatal Medicine. 2020. doi: 10.1080/14767058.2020.1763946 32419558

[28] Ceulemans M, Foulon V, Ngo E, Panchaud A, Winterfeld U, Pomar L. Mental health status of pregnant and breastfeeding women during the COVID-19 pandemic—A multinational cross-sectional study. AOGS. 2021; 00:0–11. doi: 10.1111/aogs.14092 33475148 PMC8014496

[29] López-Morales H, del Valle MV, Canet-Juric L, Andrés ML, Galli JI, Poó F, Urquijo S. Mental health of pregnant women during the COVID-19 pandemic: A longitudinal study. Psychiatry Research. 2021; 295:113567. doi: 10.1016/j.psychres.2020.113567 33213933 PMC7657008

[30] Zanardo V, Manghina V, Gilliberti L, Vettore M, Severino L, Straface G. Psychological impact of COVID-19 quarantine measures in northeastern Italy on mothers in the immediate postpartum period. Gynecology & Obstetrics. 2020. 150(2), 184–188. doi: 10.1002/ijgo.13249 32474910 PMC9087548

[31] Fallon V, Davies SM, Silverio SA, Jackson L, De Pascalis L, Harrold JA. Psychosocial experiences of postnatal women during the COVID-19 pandemic. A UK-wide study of prevalence rates and risk factors for clinically relevant depression and anxiety, Journal of Psychiatric Research. 2021, 136:157–166. doi: 10.1016/j.jpsychires.2021.01.048 33596462 PMC8635302

[32] Pariente G, Wisşotzky Broder O, Sheiner E, Lanxner Battat T, Mazor E, Salem SY. Risk for probable post-partum depression among women during the COVID-19 pandemic. Archives of Women’s Mental Health. 2020; 23:767–773. doi: 10.1007/s00737-020-01075-3 33047207 PMC7549733

[33] Silverman ME, Medeiros C, Burgos L. Early pregnancy mood before and during COVID-19 community restrictions among women of low socioeconomic status in New York City: a preliminary study. Archives of Women’s Mental Health. 2020; 2:779–782. 10.1007/s00737-020-01061-9 PMC7447087 32844329

[34] Silverman ME, Burgos L, Rodriguez ZI, Afzal O, Kalishman A, Callipari F. Postpartum mood among universally screened high and low socioeconomic status patients during COVID-19 social restrictions in New York City. Scientif reports. 2020; 10:22380. doi: 10.1038/s41598-020-79564-9 33361797 PMC7759569

[35] Kalcev G, Preti A, Orrù G, Carta MG. 2020. Perinatal Mental Health: One of the Biggest Challenges in Coronavirus Disease-19 Crisis. Open Access Maced J Med Sci. 2020. [Internet]. 8(T1), 245–7. https://oamjms.eu/index.php/mjms/article/view/5058 (assessed 18 April 2021).

[36] Davenport MH, Meyer S, Meah VL, Strynadka MC, Khurana R. Moms Are Not OK: COVID-19 and Maternal Mental Health. Frontiers in Global Women’s Health. 2020; 1. doi: 10.3389/fgwh.2020.00001 34816146 PMC8593957

[37] Spijker J, Bockting C, Meeuwissen J, van Vliet I, Emmelkamp P, Hermens M. 2013. Multidisciplinaire Richtlijn Depressie (Multidisciplinary Guideline Depression), Trimbos Institute, Utrecht, Netherlands.

[38] Donker T, van Straten A, Marks I, Cuijpers P. Quick and easy self-rating of Generalized Anxiety Disorder: validity of the Dutch-web-based GAD-7, GAD-2 and GAD-SI. Psychiatry Res. 2011; 188(1):56–64. doi: 10.1016/j.psychres.2011.01.016 21339006

[39] Pop V, Komproe I, van Son M. Characteristics of the Edinburgh postnatal depression scale in the Netherlands. Journal of Affective Disorders. 1992; 26(2):105–110. doi: 10.1016/0165-0327(92)90041-4 1447427

[40] Whooley M, Avins A, Miranda J, Browner W. Case-finding instruments for depression: two questions are as good as many, Journal of General Internal Medicine. 1997; 12 (7):439–45. doi: 10.1046/j.1525-1497.1997.00076.x 9229283 PMC1497134

[41] Spitzer RL, Williams JBW, Kroenke K, Linzer M, deGruy FV, Hahn SR. Utility of a new procedure for diagnosing mental disorders in primary care: ThePRIME-MD 1000 study. JAMA. 1994; 272(22):1749–1756. 7966923

[42] Fontein-Kuipers Y, Jomeen J. Validity and accuracy of the Whooley questions to identify maternal distress in Dutch pregnant women. The Journal of Mental Health Training, Education and practice. 2018. doi: 10.1108/JMHTEP-06-2018-0034

[43] Kroenke K, Spitzer RL, Williams JB, et al. Anxiety disorders in primary care: prevalence, impairment, comorbidity, and detection. Ann Intern Med. 2007; 146:317–25 doi: 10.7326/0003-4819-146-5-200703060-00004 17339617

[44] Nath S, Ryan EG, Trevillion K, Bick D, Demilew J, Milgrom J. Prevalence and identification of anxiety disorders in pregnancy: the diagnostic accuracy of the two-item Generalised Disorder scale (GAD-2). BMJ Open. 2018; 8:e023766. doi: 10.1136/bmjopen-2018-023766 30185582 PMC6129087

[45] Murray D, Cox J. Screening for depression during pregnancy with the Edinburgh depression scale (EPDS), Journal of Reproductive and Infant Psychology. 1990; 8(2):99–107.

[46] Fontein-Kuipers Y, Ausems M, de Vries R, Nieuwenhuijze M. The effect of Wazzup Mama?! An antenatal intervention to prevent or reduce maternal distress in pregnancy. Archives of Women’s Mental Health. 2016; 19(5):779–788. doi: 10.1007/s00737-016-0614-8 26965708

[47] Paul E, Pearson RM. Depressive symptoms measured using the Edinburgh Postnatal Depression Scale in mothers and partners in the ALSPAC Study: A date note. Welcome Open Res. 2020; 25(5):108. 10.12688/wellcomeopenres.15925.1 PMC7385546 32766456

[48] Bergink V, Kooistra L, Lambregtse-van den Berg MP, Wijnen H, Bunevicius R, van Baar A. Validation of the Edinburgh Depression Scale during pregnancy. J Psychosom Res. 2011; 70(4):385–389. doi: 10.1016/j.jpsychores.2010.07.008 21414460

[49] Levis B, Negeri Z, Sun Y, Benedetti A, Thombs B. DEPRESsion Screening Data (DEPRESSD) EPDS Group. Accuracy of the Edinburgh Postnatal Depression Scale (EPDS) for screening to detect major depression among pregnant and postpartum women: a systematic review and meta-analysis of individual participant data. BMJ. 2020; 371:m4022 doi: 10.1136/bmj.m4022 33177069 PMC7656313

[50] Harville EW, Xiong X, Buekens P, Pridjian G, Elkind-Hirsch K. Resilience after Hurricane Katrina among pregnant and postpartum women. Womens Health Issues. 2010; 20(1):20–27. doi: 10.1016/j.whi.2009.10.002 20123173 PMC2822707

[51] Matthies LM, Müller M, Doster A, Sohn C, Wallwiener M, Reck C. Maternal-fetal attachment protects against postpartum anxiety: the mediating role of postpartum bonding and partnership satisfaction. Arch Gynecol Obstet. 2020; 301(1):107–117. doi: 10.1007/s00404-019-05402-7 31875254

[52] Sibley CG, Greaves LM, Satherley N, Wilson MS, Overall NC, Lee CHJ. Effects of the COVID-19 pandemic and nationwide lockdown on trust, attitudes toward government, and well-being. American Psychologist. 2020; 75(5):618–630. doi: 10.1037/amp0000662 32496074

[53] Gao M, Hu J, Yang L, Ding N, Wei X, Li L. Association of sleep quality during pregnancy with stress and depression: a prospective birth cohort study in China. BMC Pregnancy Childbirth. 2019; 19(1):444. doi: 10.1186/s12884-019-2583-1 31775666 PMC6882237

[54] Yang X, Song B, Wu A, Mo PKH, Di J, Wang Q. Social, cognitive, and eHealth mechanisms of COVID-19-related lockdown and mandatory quarantine that potentially affect the mental health of pregnant women in China: Cross-sectional survey study. J Med Internet Res. 2021; 23(1):e24495. doi: 10.2196/24495 33302251 PMC7836909

[55] Matvienko-Sikar K, Pope J, Cremin A, Carr H, Leitao S, Olander EK. Differences in levels of stress, social support, health behaviours, and stress-reduction strategies for women pregnant before and during the COVID-19 pandemic, and based on phases of pandemic restrictions, in Ireland. Women and Birth. 2020. doi: 10.1016/j.wombi.2020.10.010 33162362 PMC7584422

[56] Ghorayeb J, Branney P, Selinger CP. When your pregnancy echoes your illness: Transition to motherhood with inflammatory bowel disease. Qualitative Health Research. 2018; 28(8):1283–1294. doi: 10.1177/1049732318763114 29577847

[57] Heiman T. Parents of children with disabilities: Resilience, coping, and future expectations. Journal of Developmental and Physical Disabilities. 2002; 14:159–171.

[58] Fergusson DM, Horwood LJ, Boden JM, Mulder RT. Impact of a major disaster on the mental health of a well-studied cohort. JAMA Psychiatry. 2014; 71(9):1025–1031. doi: 10.1001/jamapsychiatry.2014.652 25028897

